# Herpes Virus Infections in Kidney Transplant Patients (HINT) – a prospective observational cohort study

**DOI:** 10.1186/s12879-023-08663-5

**Published:** 2023-10-16

**Authors:** Sebastian Rask Hamm, Sunil Kumar Saini, Annemette Hald, Anna V. Vaaben, Natasja Wulff Pedersen, Moises Alberto Suarez-Zdunek, Zitta Barrella Harboe, Helle Bruunsgaard, Isik Somuncu Johansen, Carsten Schade Larsen, Claus Bistrup, Henrik Birn, Søren Schwartz Sørensen, Sine Reker Hadrup, Susanne Dam Nielsen

**Affiliations:** 1 Viro-Immunology Research Unit, Department of Infectious Diseases, Copenhagen University Hospital - Rigshospitalet, Copenhagen, Denmark; 2 Department of Health Technology, Technical University of Denmark, Kongens Lyngby, Denmark; 3 Department of Pulmonary and Infectious Diseases, Copenhagen University Hospital, North Zealand, Hillerød, Denmark; 4 Department of Clinical Medicine, Faculty of Health and Medical Sciences, University of Copenhagen, Copenhagen, Denmark; 5 Department of Clinical Immunology, Copenhagen University Hospital – Rigshospitalet, Copenhagen, Denmark; 6 Department of Infectious Diseases, Odense University Hospital, Odense, Denmark; 7 Department of Clinical Research, University of Southern Denmark, Odense, Denmark; 8 Department of Infectious Diseases, Aarhus University Hospital, Aarhus, Denmark; 9 Department of Nephrology, Odense University Hospital, Odense, Denmark; 10 Department of Renal Medicine, Aarhus University Hospital, and Departments of Clinical Medicine and Biomedicine, Aarhus University, Aarhus, Denmark; 11 Department of Nephrology, Copenhagen University Hospital – Rigshospitalet, Copenhagen, Denmark; 12 Department of Surgical Gastroenterology, Copenhagen University Hospital - Rigshospitalet, Copenhagen, Denmark

**Keywords:** Varicella zoster virus, Kidney transplant recipients, Kidney transplant candidates, Immune response, Herpes zoster, Shingles, Humane herpes virus 3, Shingrix, Adjuvanted recombinant glycoprotein E subunit herpes zoster vaccine, T-Lymphocytes, Immunodominant epitopes

## Abstract

**Background:**

Kidney transplant recipients receive maintenance immunosuppressive therapy to avoid allograft rejection resulting in increased risk of infections and infection-related morbidity and mortality. Approximately 98% of adults are infected with varicella zoster virus, which upon reactivation causes herpes zoster. The incidence of herpes zoster is higher in kidney transplant recipients than in immunocompetent individuals, and kidney transplant recipients are at increased risk of severe herpes zoster-associated disease. Vaccination with adjuvanted recombinant glycoprotein E subunit herpes zoster vaccine (RZV) prevents herpes zoster in older adults with excellent efficacy (90%), and vaccination of kidney transplant candidates is recommended in Danish and international guidelines. However, the robustness and duration of immune responses after RZV vaccination, as well as the optimal timing of vaccination in relation to transplantation remain unanswered questions. Thus, the aim of this study is to characterize the immune response to RZV vaccination in kidney transplant candidates and recipients at different timepoints before and after transplantation.

**Methods:**

The Herpes Virus Infections in Kidney Transplant Patients (HINT) study is a prospective observational cohort study. The study will include kidney transplant candidates on the waiting list for transplantation (*n* = 375) and kidney transplant recipients transplanted since January 1, 2019 (*n* = 500) from all Danish kidney transplant centers who are offered a RZV vaccine as routine care. Participants are followed with repeated blood sampling until 12 months after inclusion. In the case of transplantation or herpes zoster disease, additional blood samples will be collected until 12 months after transplantation. The immune response will be characterized by immunophenotyping and functional characterization of varicella zoster virus-specific T cells, by detection of anti-glycoprotein E antibodies, and by measuring cytokine profiles.

**Discussion:**

The study will provide new knowledge on the immune response to RZV vaccination in kidney transplant candidates and recipients and the robustness and duration of the response, potentially enhancing preventive strategies against herpes zoster in a population at increased risk.

**Trial registration:**

ClinicalTrials.gov (NCT05604911).

**Supplementary Information:**

The online version contains supplementary material available at 10.1186/s12879-023-08663-5.

## Background

For a selected group of patients with chronic kidney failure, kidney transplantation is the best treatment modality. After transplantation, kidney transplant recipients receive life-long immunosuppressive maintenance therapy to avoid rejection of the transplanted organ, resulting in an increased risk of infections and infection-related morbidity and mortality [1].

Varicella zoster virus (VZV) causes varicella during primary infection and herpes zoster when reactivated. In Denmark, primary vaccination against VZV is not recommended by health authorities [2], and varicella is a common childhood infection. Thus, 98% of solid organ transplant candidates in Denmark are sero-positive towards VZV [3] indicating a prior primary VZV infection. To avoid herpes zoster, cellular immunity towards VZV is important [4, 5]. However, the main target of immunosuppressive maintenance therapy post-transplantation is T cells, thus hampering the cellular immunity. The crude incidence of herpes zoster in kidney transplant recipients has been reported to be 6.7% [6], and kidney transplant recipients are at higher risk of herpes zoster than immunocompetent individuals [7–9]. Furthermore, kidney transplant recipients are at increased risk of severe herpes zoster disease and post-herpetic neuralgia [9–11].

Shingrix® (GlaxoSmithKline) is an adjuvanted recombinant glycoprotein E subunit herpes zoster vaccine (RZV). RZV prevents herpes zoster in older adults with 90% efficacy [12, 13]. A randomized clinical trial reported 68% RZV efficacy in hematopoietic stem cell transplant recipients [14], but the efficacy of RZV in kidney transplant recipients has not been established. RZV has been reported to be immunogenic and safe in kidney transplant recipients [15, 16], and RZV vaccination of kidney transplant candidates and recipients is recommended in both international and Danish guidelines [17, 18]. Although it has been suggested that cellular immunity towards VZV after natural infection and live attenuated vaccination persist over the course of transplantation [19, 20], the robustness and duration of immune responses after RZV vaccination in kidney transplant candidates and recipients, as well as the optimal timing of vaccination has not been established. Thus, characterization of immune responses to herpes zoster infection at different time points in relation to kidney transplantation might enable better preventive strategies in a population at high risk.

### Study objectives

In kidney transplant candidates and recipients receiving RZV at different time points before and after transplantation we aim to determine:

#### Primary aim

The T cell immunity towards VZV determined by deciphering the size, specificity and fitness of the T cell response following RZV, and the protective role towards herpes zoster.

#### Secondary aims

The anti-glycoprotein E antibody response rate and risk factors for anti- glycoprotein E antibody non-response.

The persistence of anti-glycoprotein E antibody and T cell immune responses.

## Methods

The Herpes Virus Infections in Kidney Transplant Patients (HINT) study is a prospective observational cohort study. Kidney transplant recipients transplanted after January 1, 2019, and kidney transplant candidates on the waiting list for kidney transplantation at Copenhagen University Hospital – Rigshospitalet, Odense University Hospital, or Aarhus University Hospital who are offered a RZV vaccine will be invited to participate in the study until February 1, 2025. Recruitment of participants started January 16, 2023 and is currently ongoing.

The kidney transplant centers at Copenhagen University Hospital – Rigshospitalet, Odense University Hospital, or Aarhus University Hospital are responsible for the inclusion and follow-up of participants, while researchers from the department of health technology at Technical University of Denmark (DTU) are responsible for the immunological analyses. The study is performed in collaboration with the herpesvirus immunology in solid organ transplant recipients – liver transplant study (HISTORY) and parts of the methodology follows what has previously been described in the HISTORY study protocol [21]. A STROBE [22] checklist is provided as supplementary material (supplementary material).

### Vaccinations

All participants who accept the offer to receive RZV vaccination are vaccinated according to Danish guidelines at their local transplant center. Therefore, vaccinations are performed uniformly at all three centers by administering two doses of vaccine with two months between each dose. All vaccinations administered are a part of routine clinical care.

### Eligibility criteria

Participants must be either on the waiting list for kidney transplantation OR first time transplanted after January 1, 2019, AND above 18 years old, able to provide informed consent, and have been offered a RZV vaccine. Patients are eligible to participate whether they accept RZV vaccination or not.

### Blood sampling

Venous blood samples are collected from participants at scheduled visits to the clinical sites. All participants will provide a blood sample at inclusion i.e., at the time they are offered the first dose of RZV vaccination. Hereafter, all participants will be invited for repeated blood sampling 1, 2, 6, and 12 months after the first dose of RZV was offered. Furthermore, participants who are on the waiting list for transplantation and transplanted during the study will be invited for blood sampling at 6 and 12 months after transplantation. If a participant develops herpes zoster during the study period, they will be invited for blood sampling immediately after the infection has been diagnosed. At each sample time, 60 mL of venous blood will be collected in lithium-heparin and EDTA-coated collection tubes.

Within 4 h after collection, blood collected in lithium-heparin-coated collection blood tubes will be transported to the laboratory for purification of peripheral blood mononuclear cells (PBMC) using density gradient centrifugation. EDTA-coated collection tubes will be stored on ice immediately after collection and centrifuged within 1 h, after which plasma will be separated and stored at -80 °C. After primary processing at either Copenhagen University Hospital – Rigshospitalet, Odense University Hospital, or Aarhus University Hospital, all PBMC and plasma samples will be stored in liquid nitrogen at a biobank at the Department of Health Technology, DTU.

### Questionnaire

At inclusion, participants will be asked to fill out a questionnaire about health, lifestyle, socioeconomic status, current and previous use of medication and vaccinations. The questionnaire will be entered directly into REDCap [23]—a secured digital platform or on paper (supplementary material). At each following study visit, participants will be asked to fill out a questionnaire regarding changes related to transplantation, vaccinations, rejection episodes, and infections since the last study visit (supplementary material).

### Antibody assay

The anti-glycoprotein E antibody response rate will be determined by Enzyme-Linked Immunosorbent Assay (ELISA). Glycoprotein E is the main target for VZV-specific immune responses [24]. We will establish and validate a previously reported ELISA and measure anti-glycoprotein E antibody concentrations in plasma samples collected from all participants at all time points. The anti-glycoprotein E antibody response rate will be defined as the percentage of subjects with anti-glycoprotein E antibody concentration ≥ 4-fold higher than at baseline.

### Identification of VZV specific T cells

**Fig. 1:**
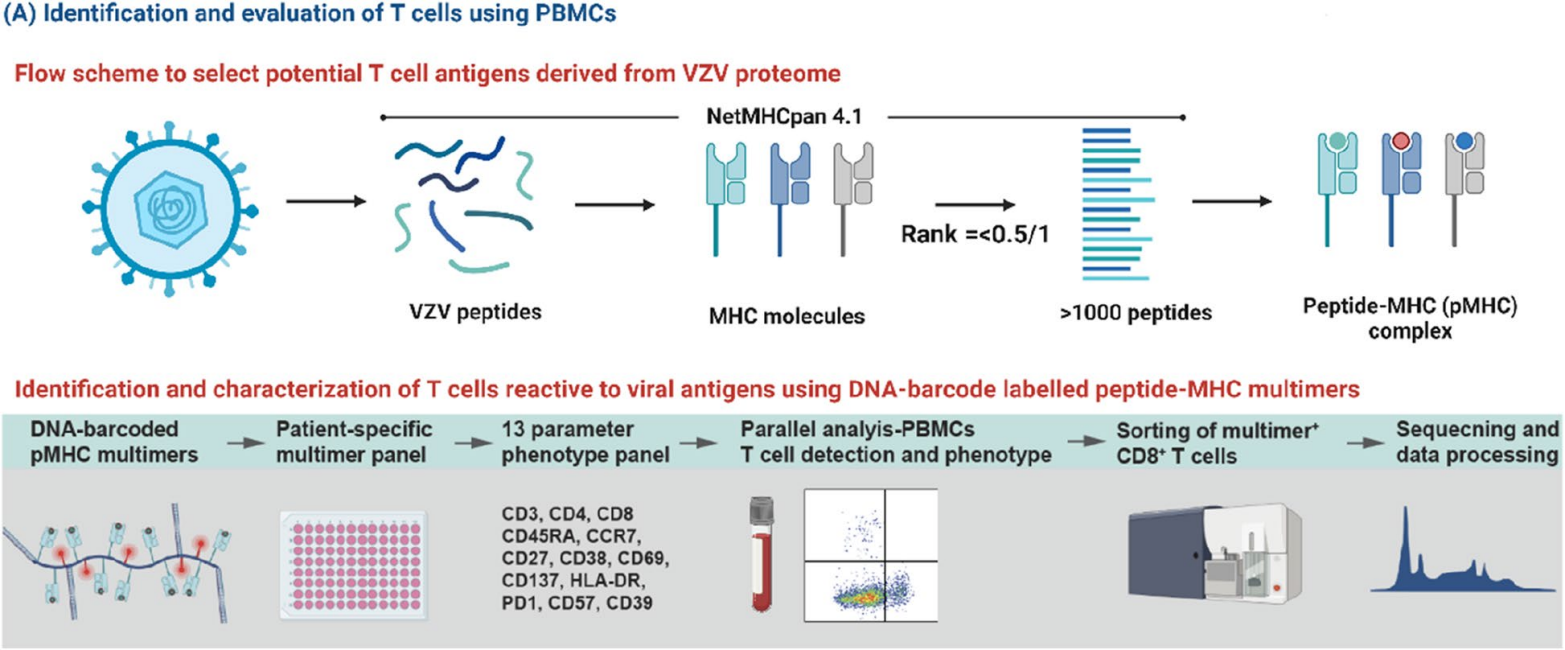
Overview of methods for identification and evaluation of T cells using peripheral blood mononuclear cells

To identify the complete repertoire of VZV-specific T cells and their immunodominance towards VZV, we will perform a broad T cell screening towards potential T cell epitopes in VZV, as previously described [21]. Human leukocyte antigen (HLA)-class I presented epitopes will be predicted (NetMHCpan-4.1) and synthetized to make peptide-HLA multimers matching the HLA profile of the individual participant (Fig. 1). We will cover the 12 most frequent HLA haplotypes [25]. Using DNA-barcode labeled peptide-major histocompatibility complex (pMHC) multimers and subsequent flow cytometry-based sorting of VZV-specific T cells, we can determine the T cell recognition to a large library of potential epitopes (> 1000/sample) [26, 27]. The sequential sampling pre- and post-transplantation will allow a comparative assessment and monitoring of T cell frequency, phenotype and fitness at different levels of immunosuppression.

Using the NetMHCpan 4.1 platform, as previously described in the HISTORY study protocol [21], potential varicella-zoster virus (VZV) peptide-MHC (pMHC) complexes will be predicted corresponding to the most frequent HLA haplotypes amongst participants. The pMHC complexes will be combined in a DNA-barcoded patient-specific pMHC multimer. Peripheral blood mononuclear cells (PBMCs) will be examined for their detection of pMHC multimers and sorted using flow cytometry based on their expression of phenotypic cell surface markers. The reactive T cells may be further examined for their frequency, T cell receptor sequence, and potential for ex vivo expansion [21].

### Phenotype and cytokine profiling

**Fig. 2:**
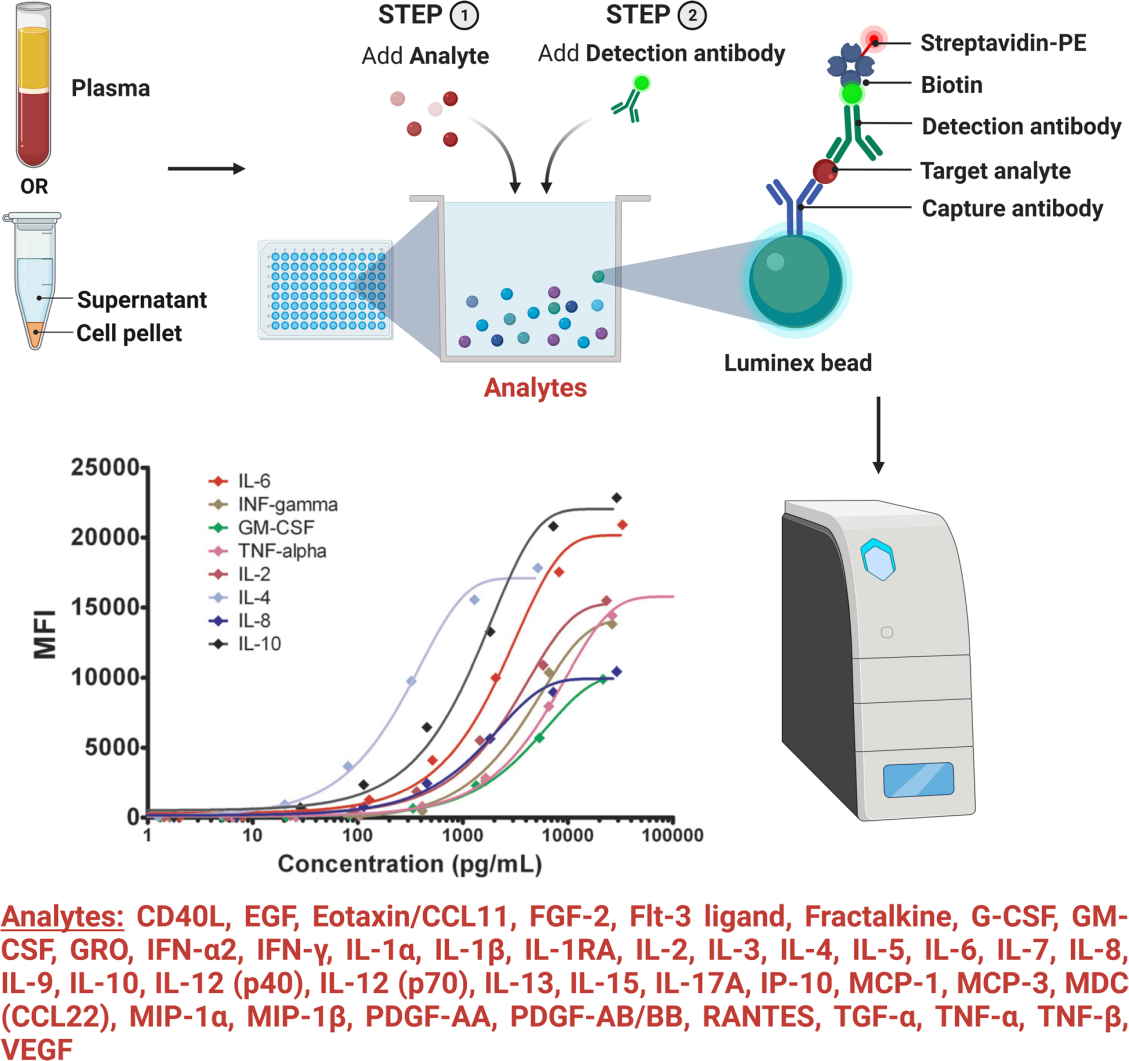
Overview of methods for cytokine profiling using Luminex MILLIPLEX® assay

Phenotyping and functional characterization of specific T cell populations towards immunodominant epitopes in VZV will be carried out, as previously described in the HISTORY study protocol [21], by using a flow cytometry panel of 13 cell surface and intracellular markers of activation, senescence, and exhaustion (Fig. 1). Furthermore, we will measure the cytokine profiles in plasma samples using an established Luminex MILLIPLEX assay to simultaneously quantify 41 analytes that map the complete cytokine signature including interleukins, chemokines, and growth factors (Fig. 2).

Cytokine profiling will be performed as previously described in the HISTORY study protocol [21]. Plasma contains an unspecified concentration of analytes of interest, i.e. cytokines, chemokines, interleukins and growth factors. To determine the concentration of the analytes, each Luminex well contains multiple types of color-coded beads conjugated to a capture antibody that binds the specific analyte of interest. In presence of the analyte, a biotin-conjugated detection antibody will bind to the capture antibody-analyte complex. Adding phycoerythrin-labeled streptavidin will produce fluorescence that is color-specific for each analyte and allows for measurement of the mean fluorescence intensity (MFI) of each analyte in each Luminex well. Comparing MFI at different dilution levels with standard solutions allows for determination of the concentration of the analytes of interest.

### T cell receptor sequencing

We will apply a technology that allows for large-scale collection of T cell receptor (TCR) sequences paired to different peptide targets based on their pMHC recognition motif through a pMHC multimer linked to a DNA barcode [28]. Using single-cell capture and analysis systems based on sorted pMHC responsive T cells, we can pair the TCR sequences and assign the pMHC specificity based on the co-attached DNA barcode. Using this platform, we will identify T cell clonotypes of relevance for VZV recognition and describe their functional characteristics. We will implement parallel phenotyping of immune cells using a 150 + marker panel of DNA barcoded antibodies that target a broad array of lineage markers, activation markers, exhaustion markers, and regulatory markers.

### Clinical data

Data on demographics, comorbidities, biochemistry, medication, allograft rejection, pathology, HLA, and hospital admissions will be collected from electronical hospital records. All Danish hospital records since 1 December 2010 are accessible through a nationwide digital platform. Data on vaccinations will be collected through the Danish vaccination register (DDV) [29]. Since 2015, it has been mandatory to register all vaccinations administered in Denmark in DDV. Data on infections will be collected from the Danish Microbiology database (MiBa) [30], which is a nationwide database including all microbiological samples collected at both hospitals and general practitioners in Denmark.

### Statistics

#### Sample size

The purpose of the study is to describe the T-cell and anti-glycoprotein E antibody response to RZV and herpes zoster in kidney transplant candidates and recipients and to explore factors for immune non-response at different times before and after transplantation. In Denmark about 390 patients are active on the waiting list for kidney transplantation and about 250 kidney transplantations are performed each year [31]. We aim to include 250 kidney transplant recipients who are 6–12 months from transplantation, 125 who are 12–24 months from transplantation, and 125 who are > 24 months. Furthermore, since some of the transplant candidates may not be transplanted during the study period, we will include 375 patients who are on the waiting list. Given the descriptive and exploratory scope of the study and lack of existing data regarding outcomes of interest, a formal sample size calculation cannot be conducted. The study is not powered for clinical efficacy estimates.

#### Statistical analyses

Continuous data will be compared using Student’s t test or Mann–Whitney U test, and categorical data will be compared using Pearson’s χ^2^ or Fisher’s exact tests, as appropriate. Mixed linear models will be used to evaluate the development of antibody and T cell immunity towards VZV over time and to explore risk factors for non-response.

## Discussion

Kidney transplant recipients are at higher risk of herpes zoster than immunocompetent individuals [7–9] and at increased risk of severe herpes zoster disease and post-herpetic neuralgia [9–11]. Current international and Danish guidelines recommend RZV vaccination of kidney transplant candidates prior to transplantation [17, 18]. However, evidence on the robustness and duration of the immune response, as well as the optimal timing of vaccination is warranted. Through a characterization of the VZV specific immune response in kidney transplant candidates and recipients, this study has the potential to improve preventive strategies against herpes zoster in a population at risk. By observing immune responses in kidney transplant candidates and recipients vaccinated at different time points with regards to transplantation we expect to be able to establish the most optimal timing of vaccination for kidney transplant candidates and recipients. Furthermore, identification of risk factors for immune non-response could provide guidance for increased surveillance of individuals at higher risk of herpes zoster and a potential need for additional RZV booster doses.

### Supplementary Information

**Additional file 1. **

**Additional file 2. **

**Additional file 3. **

## Data Availability

The datasets generated and/or analyzed during the current study are not publicly available due privacy regulations but are available from the corresponding authors on reasonable request.

## Abbreviations

HINT: Herpesvirus Infections in Kidney Transplant Patients Study
VZV: Varicella zoster virus
HZ: Herpes zoster
RZV: Adjuvanted recombinant glycoprotein E subunit herpes zoster vaccine
ELISA: Enzyme-Linked Immunosorbent Assay
PBMC: Peripheral blood mononuclear cells
pMHC: Peptide-Major Histocompatibility Complex
HLA: Human leukocyte antigen
TCR: T cell receptor
DDV: The Danish vaccination register
MiBa: The Danish microbiology database
