# Supplementary material for: Herpes Virus Infections in Kidney Transplant Patients (HINT) – a prospective observational cohort study
Source: BMC Infect Dis. 2023 Oct 16;23:687. doi: 10.1186/s12879-023-08663-5 (PMC10578002; doi:10.1186/s12879-023-08663-5)
Supplement: Supplementary file 2 — Additional file 2. [file 12879_2023_8663_MOESM2_ESM.docx]

HINT-ID no.:

(filled out by study personnel)

HINT-STUDY

**QUESTIONNAIRE 1**

In this questionnaire we will ask you to answer some questions about your heath and lifestyle. **We ask you to answer all questions.** The questions are answered by ticking the box that is most appropriate. Your answers will be treated with **strict confidentiality**

| Name |  | | |
| --- | --- | --- | --- |
| Address |  | | |
| Postal code & city |  | | |
| Telephone no. |  | CPR-no |  |
| E-mail |  | | |

**Vaccination history**

In the following we ask you to state which vaccines you have received since your last study visit:

| Yes | | No | Un-known | Date : | If known: What type of vaccine? |
| --- | --- | --- | --- | --- | --- |
| Tuberkulose | □ | □ | □ |  |  |
| Human papillomavirus (HPV) | □ | □ | □ |  |  |
| Haemophilus influenzae type B (Hib) | □ | □ | □ |  |  |
| Chickenpox/shingles (varicella-zoster) | □ | □ | □ |  |  |
| Hepatitis A | □ | □ | □ |  |  |
| Hepatitis B | □ | □ | □ |  |  |
| Influenza (1^st^ time) | □ | □ | □ |  |  |
| “ (2^nd^ time) | □ | □ | □ |  |  |
| “ (3^rd^ time) | □ | □ | □ |  |  |
| “ (more than 3) | □ | □ | □ |  |  |
| Pneumococcal disease (including pneumonia) | □ | □ | □ |  |  |
| COVID-19 | □ | □ | □ |  |  |
| Other | □ | □ | □ |  |  |

|  |  |  |  |  |  |
| --- | --- | --- | --- | --- | --- |

**Infections**

**Herpes infections**

| Have you had a herpes infections since your last study visit? Yes: □ No: □ |  |
| --- | --- |
| **If yes:** Which treatment did you recieve?  ___________________________________________ __________________  **If yes:** Was an extra blood sample collected for this project? Yes: □ No: □ | |

**Other infections**

Have you since your last visit had any other infections (Not including herpes)? Yes: □No: □

**If yes:** Which infection? __________________________________________________ _

**If yes:** Which treatment did you receive?

_______________________________________________________________ _

**Transplantation related changes**

**Transplantation**

Have you recieved a kidney transplant since your last visit? Yes: □ No: □

**If yes:** Which date? ______________________

**Rejection**

Have you had an allograft rejection episode since your last visit? Yes: □ No: □

**If yes:** Which treatment did you receive?

_______________________________________________________________ _
