# Supplementary material for: Herpes Virus Infections in Kidney Transplant Patients (HINT) – a prospective observational cohort study
Source: BMC Infect Dis. 2023 Oct 16;23:687. doi: 10.1186/s12879-023-08663-5 (PMC10578002; doi:10.1186/s12879-023-08663-5)
Supplement: Supplementary file 3 — Additional file 3. [file 12879_2023_8663_MOESM3_ESM.docx]

HINT-ID no.:

(filled out by study personnel)

HINT-STUDY

**QUESTIONNAIRE 1**

In this questionnaire we will ask you to answer some questions about your heath and lifestyle. **We ask you to answer all questions.** The questions are answered by ticking the box that is most appropriate. Your answers will be treated with **strict confidentiality**

| Name |  | | |
| --- | --- | --- | --- |
| Address |  | | |
| Postal code & city |  | | |
| Telephone no. |  | CPR-no |  |
| E-mail |  | | |

**Vaccination history**

In the following we ask you to state which vaccines you have received:

| Yes | | No | Un-known | Approx year: | If known: What type of vaccine? |
| --- | --- | --- | --- | --- | --- |
| Tuberkulose | □ | □ | □ |  |  |
| Human papillomavirus (HPV) | □ | □ | □ |  |  |
| Haemophilus influenzae type B (Hib) | □ | □ | □ |  |  |
| Chickenpox/shingles (varicella-zoster) | □ | □ | □ |  |  |
| Hepatitis A | □ | □ | □ |  |  |
| Hepatitis B | □ | □ | □ |  |  |
| Influenza (1^st^ time) | □ | □ | □ |  |  |
| “ (2^nd^ time) | □ | □ | □ |  |  |
| “ (3^rd^ time) | □ | □ | □ |  |  |
| “ (more than 3) | □ | □ | □ |  |  |
| Pneumococcal disease (including pneumonia) | □ | □ | □ |  |  |
| COVID-19 | □ | □ | □ |  |  |
| Other | □ | □ | □ |  |  |

**Helbreds- og livsstilsspørgeskema**

In the following we kindly ask you to respond to questions regarding your lifestyle and health. Please respond to the best of your ability. If you do not know the exact year, please provide the best approximate.

|  |  | Yes | No |
| --- | --- | --- | --- |
| 1. | Do you experience pain or tightness in your chest when you are in a hurry, or when you walk up stairs? | □ | □ |
| 2. | Do you have shortness of breath when you are in a hurry or go up a hill? | □ | □ |
| 3. | Do you have more shortness of breath when walking at normal pace on a straight road compared to people your own age? | □ | □ |
| 4. | Do you sometimes have to stop and catch your breath when walking down the street at your own pace? | □ | □ |
| 5. | Do you sometimes wake up at night due to shortness of breath or strenuous breathing? | □ | □ |
| 6. | Do you have shortness of breath when taking a bath or when getting dressed? | □ | □ |
| 7. | Do you often experience shortness of breath when you wake up in the morning? | □ | □ |
| 8. | Do you have shortness of breath when sitting quietly or resting? | □ | □ |
| 9. | Have you coughed persistently for the past 8 weeks? | □ | □ |
| 10. | Do you cough up mucus (in the mornings or during the day) as long as 3 consecutive months a year? | □ | □ |
|  | **If yes**: Has this been going on for 2 consecutive years or more? | □ | □ |

|  |  | | | | | | | | | | | | yes | | No | |  |
| --- | --- | --- | --- | --- | --- | --- | --- | --- | --- | --- | --- | --- | --- | --- | --- | --- | --- |
| 12. | | Have you been exposed to dust or fumes over long periods of time in your working life?  **If yes:** How many years? Number of years: _ | | | | | | | | | | | □ | | □ | | |
| 13. | | Do you sometimes experience wheezing (highpitched whistling sound during breathing)? | | | | | | | | | | | □ | | □ | | |
|  | | **Hvis ja:** During a cold? | | | | |  |  |  |  | |  | □ | | □ | | |
|  | |  | During physical activity? | | | |  |  |  |  | |  | □ | | □ | | |
|  | |  | Without known cause? | |  |  |  |  |  |  | |  | □ | | □ | | |
| 21. | | Have you had an episode of acute fever in the last four weeks? | | | | | | | | | | | □ | | □ | | |
| 24. | | Do you smoke? | | | | | | | | | | | □ | | □ | | |
|  | | **If no**: Have you previously smoked  If you have never smoked, please go to question 29. | | | | | | | | | | | □ | | □ | | |
| 25. | | How many years have you smoked? Number of years | | | | | | | | |  | | | | | |  |
| 26. | | How old were you when you started smoking? år | | | | | | | | |  | | | | | |  |
| 27. | | **If you used to smoke,** how old were you when you stoped smoking? år | | | | | | | | | | | | | | |  |
| 28. | | If you smoke or have previously smoked, how much is/was you average consumption of: | | | | | | | | | | | | | | |  |
|  | | Cigarettes without filter | | Quantity per day: | | | | | | |  | | | | | |  |
|  | | Cigarettes with filter | | Quantity per day: | | | | | | |  | | | | | |  |
|  | | Cheroots | | Quantity per day: | | | | | | |  | | | | | |  |
|  | | Cigars | | Quantity per day: | | | | | | |  | | | | | |  |
|  | | Pipe tobacco | | Packets of 40/50 g per week: | | | | | | | | | | | | |  |
| 34. | | How many units of alcohol do you drink **per week?**: __________ | | | | | | | | | | | | | | |  |

| 39. | Indicate your physical activity AT WORK in the last year (also filled in by housewives, students and currently unemployed, while pensioners without actual employment are asked to go to question 47.) **(Only one answer)** | | |
| --- | --- | --- | --- |
|  | 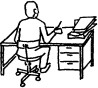I. Predominantly sitting work e.g., desk job, homemaker without children, and with a maid | | □ |
|  | 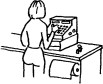II. Sitting or standing, sometimes walking e.g., clerk, teacher, homemaker who does all the washing and cleaning themselves, without small children | | □ |
|  | 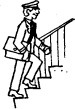  III. Walking, sometimes lifting e.g., postman, health care worker, who does all the washing and cleaning themselves, with one or more small children | | □ |
|  | 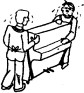  IV. Heavy bodywork e.g., movers, construction workers |  | □ |
|  | If yes to III or IV: Do you often lift heavy loads? Yes □ No □ | |  |
|  | On average, how many hours a week do you work outdoors? Hours | | |
| 40. | Indicate your physical activity DURIN LEISURE TIME (including transport to and from work) within the last year **(Only one answer)** | | |
|  | 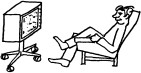  II. Almost completely physically inactive or light physical activity up to 2 hours a week. e.g., reading, television, cinema 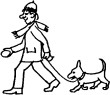 | | □ |
|  | 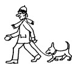  II. Light physical activity from 2 - 4 hours a week e.g., walks, biking, light gardening, light exercise | | □ |
|  | 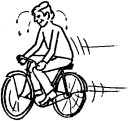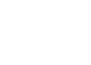III. Light physical activity for more than 4 hours per week or more intense physical activity for 2 - 4 hours per week e.g. fast walking and/or fast cycling, laborious gardening, heavy exercise with sweating or breathlessness | | □ |
|  | 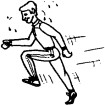IV. Intense physical activity for more than 4 hours a week or regular intense training and potentially with participation in competitions several times a week | | □ |
|  | If yes to III or IV: Does your training involve weight-lifting or heavier strength/weight? Yes □ No □ | | |
|  | On average, how many hours per week have you been outdoors in your spare time? Hours: _________ | | |

| **Socioeconomic questionnaire**  In the following, we kindly ask you to respond to questions regarding your education and demographics. | |
| --- | --- |
| 42. | What is the longest education you have completed after you left primary school? (**one X**)  No education  Short cycle higher education (e.g. academy of professional higher education)  Vocational training or similar (1-3 years)  Medium-cycle higher educaiton (e.g. teacher, nurse etc.)  Long-cycle higher educaiton  Ph.d. or equivalent |
| 44. | Do you live:  With spouse/companion  Alone  With other |
| 45. | How many people are in your household including yourself? Quantity: |
